# Interactive Effects of Temperature and UV Radiation on Photosynthesis of *Chlorella* Strains from Polar, Temperate and Tropical Environments: Differential Impacts on Damage and Repair

**DOI:** 10.1371/journal.pone.0139469

**Published:** 2015-10-01

**Authors:** Chiew-Yen Wong, Ming-Li Teoh, Siew-Moi Phang, Phaik-Eem Lim, John Beardall

**Affiliations:** 1 Department of Human Biology, International Medical University, No. 126, Jalan Jalil Perkasa 19, Bukit Jalil, 57000, Kuala Lumpur, Malaysia; 2 Institute of Ocean & Earth Sciences, University of Malaya, 50603, Kuala Lumpur, Malaysia; 3 National Antarctic Research Centre, Institute of Graduate Studies, University of Malaya, 50603, Kuala Lumpur, Malaysia; 4 School of Biosciences, Taylor’s University, Taylor’s Lakeside Campus, No. 1, Jalan Taylor’s, 47500, Subang Jaya, Selangor Darul Ehsan, Malaysia; 5 Institute of Biological Sciences, University of Malaya, 50603, Kuala Lumpur, Malaysia; 6 School of Biological Sciences, Monash University, Clayton, Victoria, 3800, Australia; Federal University of Rio de Janeiro, BRAZIL

## Abstract

Global warming and ozone depletion, and the resulting increase of ultraviolet radiation (UVR), have far-reaching impacts on biota, especially affecting the algae that form the basis of the food webs in aquatic ecosystems. The aim of the present study was to investigate the interactive effects of temperature and UVR by comparing the photosynthetic responses of similar taxa of *Chlorella* from Antarctic (*Chlorella* UMACC 237), temperate (*Chlorella vulgaris* UMACC 248) and tropical (*Chlorella vulgaris* UMACC 001) environments. The cultures were exposed to three different treatments: photosynthetically active radiation (PAR; 400–700 nm), PAR plus ultraviolet-A (320–400 nm) radiation (PAR + UV-A) and PAR plus UV-A and ultraviolet-B (280–320 nm) radiation (PAR + UV-A + UV-B) for one hour in incubators set at different temperatures. The Antarctic *Chlorella* was exposed to 4, 14 and 20°C. The temperate *Chlorella* was exposed to 11, 18 and 25°C while the tropical *Chlorella* was exposed to 24, 28 and 30°C. A pulse-amplitude modulated (PAM) fluorometer was used to assess the photosynthetic response of microalgae. Parameters such as the photoadaptive index (E_k_) and light harvesting efficiency (α) were determined from rapid light curves. The damage (*k*) and repair (*r*) rates were calculated from the decrease in ΦPSII_eff_ over time during exposure response curves where cells were exposed to the various combinations of PAR and UVR, and fitting the data to the Kok model. The results showed that UV-A caused much lower inhibition than UV-B in photosynthesis in all *Chlorella* isolates. The three isolates of *Chlorella* from different regions showed different trends in their photosynthesis responses under the combined effects of UVR (PAR + UV-A + UV-B) and temperature. In accordance with the noted strain-specific characteristics, we can conclude that the repair (*r*) mechanisms at higher temperatures were not sufficient to overcome damage caused by UVR in the Antarctic *Chlorella* strain, suggesting negative effects of global climate change on microalgae inhabiting (circum-) polar regions. For temperate and tropical strains of *Chlorella*, damage from UVR was independent of temperature but the repair constant increased with increasing temperature, implying an improved ability of these strains to recover from UVR stress under global warming.

## Introduction

The anthropogenic release of chlorofluorocarbons (CFCs) and other active compounds into the atmosphere causes the breakdown of ozone in the stratosphere and this leads to a rise in the flux of ultraviolet-B radiation (UV-B, 280–320 nm) transmitted to the Earth’s surface. This is most marked at (but is not exclusive to) high latitudes [1]. Increased global warming, leading to enhanced cooling in the stratosphere, influences the extent of ozone depletion and the consequent increases in UV-B incident on the surface of the planet [2]. Although this is expected to show a gradual recovery in years to come [3, 4], ozone depletion, exacerbated by global warming [5], is still a significant problem for many organisms.

Photoautotrophic organisms, including algae (which contribute 50% of the planet’s primary productivity [6, 7]), use solar radiation as a principal energy source to drive physiological processes such as photosynthesis and growth. However, high levels of solar ultraviolet radiation (UVR), and especially UV-B, are considered to be a stressor for many physiological processes and will cause damage to DNA [8, 9], inhibit photosynthetic rate [10, 11] and inactivate enzymes [11]. These biologically harmful effects of UVR can negatively affect the diversity and species richness of algal communities [12].

Photosystem II (PSII) is one of the main molecular targets of UV-B-induced photoinhibition in algae [13–15] through the effects of UVB on D1 and D2 proteins in the PSII reaction centre, though UV-B also affects the C-fixing enzyme Rubisco. A number of studies have revealed that the maximum quantum yield (ΦPSII_max_, = F_v_/F_m_) and the electron transport rate (ETR) of microalgae are negatively affected by UV-B radiation [16–20]. These impacts, however, are modulated by interactions between UVR and other factors such as light availability, nutrient limitation and levels of dissolved inorganic carbon, all of which are features of the environment that are likely to alter as a consequence of global change predicted for the next century and beyond. Interactions between UV-B and nutrient levels [21, 22], PAR intensity [23] and CO_2_ [24] have all been reported recently [25].

Changing atmospheric and surface sea temperatures will potentially enhance water column stratification and thereby have an impact on the nutrient status of phytoplankton, at least in the tropics and mid-latitudes [26], which in turn will exert an influence on UV-B sensitivity (see above). Extensive studies have been carried out on the independent effects of UVR and temperature on the physiology of algae [27–35]. Our study adds to the growing body of work showing interactions between temperature and UVR sensitivity by specifically examining effects of temperature on the damage and repair cycle under UVR [36–41].

The net effect of UVR on photosynthesis (P), and particularly on PSII activity, can be explained by a number of models, the simplest being the Kok model [42] in which the net impact is a balance between damage (*k*), which is a function of the number of remaining targets, and repair (*r*) at time *t*, where P_initial_ is the rate of photosynthesis before exposure commences [43] (Eq 1).

$$\frac{P}{P_{initial}}\text{=}\frac{r}{k+r}+\frac{k}{k+r}e^{-(k+r)t}$$ (1)

Since damage to PSII reaction centres is driven by photochemistry, and photochemical events are largely temperature insensitive, values of *k* should be relatively insensitive to temperature. In contrast, repair processes such as turnover and re-synthesis of D1 protein are enzymically driven, so the repair constant *r* should be strongly temperature sensitive within physiological limits.

Algae live in environments with multiple abiotic factors that change simultaneously, act in combination or are inter-dependent. An increase in UVR has been reported over the past 20 years as a result of decreased stratospheric ozone [44–46] while the average global surface temperature will increase by around 4–5°C over the next century because of global warming [47]. It is therefore important to understand possible interactions between temperature and UVR due to global warming and to estimate the possible ecological implications of such environmental changes.

The small coccoid chlorophyte, *Chlorella*, which is cosmopolitan in occurrence, is one of the best-studied phototrophic eukaryotes. It can be found in a diverse range of habitats such as in soil, freshwater lakes, ponds, marine and brackish waters, and snow as well as in hot springs [48–49]. Studies have reported that *Chlorella* strains isolated from Antarctic and Arctic are eurythermal, able to grow at temperatures ranging from 4–30°C and 3–27°C, respectively [35, 50]. *Chlorella* is an ideal experimental organism for investigating various research questions and has potential applications in biotechnology [48]. Due to the wide occurrence and applications of *Chlorella*, and the availability of strains from various thermal habitats, this alga was chosen for the present study, which seeks to explore further the question of how microalgae, especially *Chlorella*, can cope with exposure to UVR.

Here we report on studies of the interactive effects of temperature and UVR by comparing the responses of an isolate of *Chlorella* from the Antarctic to those of temperate and tropical isolates of the same genus. We test the hypothesis that *r* should exhibit a greater temperature sensitivity than *k* and that therefore higher temperatures should promote repair more than damage, with the consequence that net UVR damage should be less at higher temperatures. To this end we exposed cultures of 3 strains of *Chlorella* spp., isolates from polar, temperate and tropical freshwaters, to a range of temperatures and used PAM fluorometry to measure photosynthetic characteristics during exposure to photosynthetically active radiation (PAR, 400–700 nm), PAR plus UV-A (320–400 nm) radiation (PAR + UV-A) and PAR plus UV-A and UV-B (280–320 nm) radiation (PAR + UV-A + UV-B).

## Materials and Methods

### Ethics statement

*Chlorella* isolates were obtained from existing holdings in the University of Malaya Algae Culture Collection (UMACC). They are not endangered or protected species. No permits are required to study these algae.

### Algae cultures

Three strains of *Chlorella*, namely *Chlorella* UMACC 237, *Chlorella vulgaris* UMACC 248 and *C*. *vulgaris* UMACC 001 were used in the present study. The Antarctic *Chlorella* UMACC 237 was isolated in 2002 from a soil sample collected near a wastewater pond at Casey Station, Antarctica. The temperate *Chlorella* UMACC 248 was obtained from the Culture Collection of Algae and Protozoa (CCAP) and was originally isolated from a freshwater lake in the Netherlands in 1892, while the tropical *Chlorella* UMACC 001 was isolated from a fish pond at the University of Malaya in 1987 [32]. Although the *Chlorella* isolates used in the present study were isolated at different times, each was maintained at or close to its temperature optimum as it is known that long-term cultivation may cause accumulation of mutations and in vitro selection [51]. However, some microalgal strains may show high genetic stability despite long-term cultivation [52]. The cultures were grown in Bold’s basal medium (BBM) [53] and maintained in a controlled-environment incubator at 4, 18 or 28°C for the Antarctic, temperate and tropical *Chlorella*, respectively, illuminated with cool white fluorescent lamps (Philips, TLD 18W/54-765) providing 42 μmol m^-2^ s^-2^ PAR on a 12 h:12 h light:dark cycle. The identification of the *Chlorella* spp. from the Antarctic and tropics was based on morphological studies (unpublished data).

### Experimental design

The cultures were exposed in the incubator at different temperatures for one hour, to three light treatments: PAR + UV-A, PAR + UV-A + UV-B and PAR alone. They were irradiated with a combination of three types of lamps: two tubes of daylight fluorescent lamps (Philips, TLD 18W/54-765, Thailand) providing 42 μmol m^-2^ s^-1^ of photosynthetically active radiation (PAR), one UV-B lamp (Philips, TL 20W/12RS, Holland) providing an irradiance of 1.17 W m^-2^ and UV-A lamps (Philips, TLK 40W/10R, Holland) providing an irradiance of 8.54 W m^-2^. The emission range for the UV-B lamp is 290 to 320 nm with a peak at 302 nm while the UV-A lamp has a wavelength of between 315 to 380 nm with an emission peak at 350 nm. Details of spectra for these lamps are available at http://www.lighting.philips.com/main/prof/lamps/fluorescent-lamps. The irradiances were measured using a SpectroSense2 4-channel display meter fitted with UV-A, UV-B and PAR Quantum sensors. The UV-B dose applied was higher, and the UV-A lower, than is found in many habitats but were used to provide an acute UV stress to determine how temperature influenced the damage and repair processes [43]. Various cut-off filters were used to obtain the different UVR treatments. For the PAR alone, the cultures were grown in quartz tubes covered with polycarbonate sheet to eliminate UV-A and UV-B radiation. To obtain the PAR + UV-A treatment, Mylar sheet was used to cut off the UV-B radiation. The cultures receiving PAR + UV-A + UV-B were covered with a Whirl-Pack^R^ bag to allow the light spectrum above 280 nm to pass through. It has been shown that these bags are biologically inert and transparent to ecologically relevant UVR wavelengths [54]. The Antarctic *Chlorella* was exposed to 4, 14 and 20°C. The temperate *Chlorella* was exposed to 11, 18 and 25°C while the tropical *Chlorella* was exposed to 24, 28 and 30°C. These temperatures were chosen to represent sub-optimal, optimal and, in the case of the Antarctic isolate at 20°C, supra-optimal temperatures for the different strains [34, 35].

An Underwater Fluorometer Diving-PAM (Heinz Walz GmbH, Effeltrich, Germany) was used to measure the photosynthetic properties of the microalgae studied [55]. A rapid light curve (RLC) curve was measured at 0 h (before exposure to UVR) and after 1 h exposure to the appropriate UVR and temperature treatment. RLCs were run with actinic light intensities up to 1560 μmol m^-2^ s^-1^, with each actinic light exposure lasting 10 s. A dark adaptation period of 15 minutes before the RLC allowed determination of maximum quantum yield (ΦPSII_max_). Photosynthetic parameters such as the photoadaptive index (E_k_; a measure of the light intensity for saturation of electron transport), maximum rates of electron transport (rETR_max_), and light harvesting efficiency (α) were determined according to [55], and are related through the equation E_k_ = rETR_max_/alpha. Exposure response curves examining the effects of UVR and temperature on effective quantum yield (ΦPSII_eff_) were carried out by applying a saturation pulse every 5 minutes for 1 h for cells exposed to PAR + UV-A + UV-B, at different temperatures. The effect of temperature on damage (*k*) and repair (*r*) rates was calculated from the decrease in ΦPSII_eff_ over time by fitting the data to the Kok model (Eq 1), using GraphPad Prism.

### Statistical analysis

The means and standard deviation values of the triplicate cultures under each treatment were calculated. One-way analysis of variance (ANOVA) was used to determine whether there was any significant difference (p<0.05) between the treatments used in all the experiments, followed by comparison of means using Duncan’s test. All statistical analyses were performed using SPSS software.

## Results

**Fig 1:**
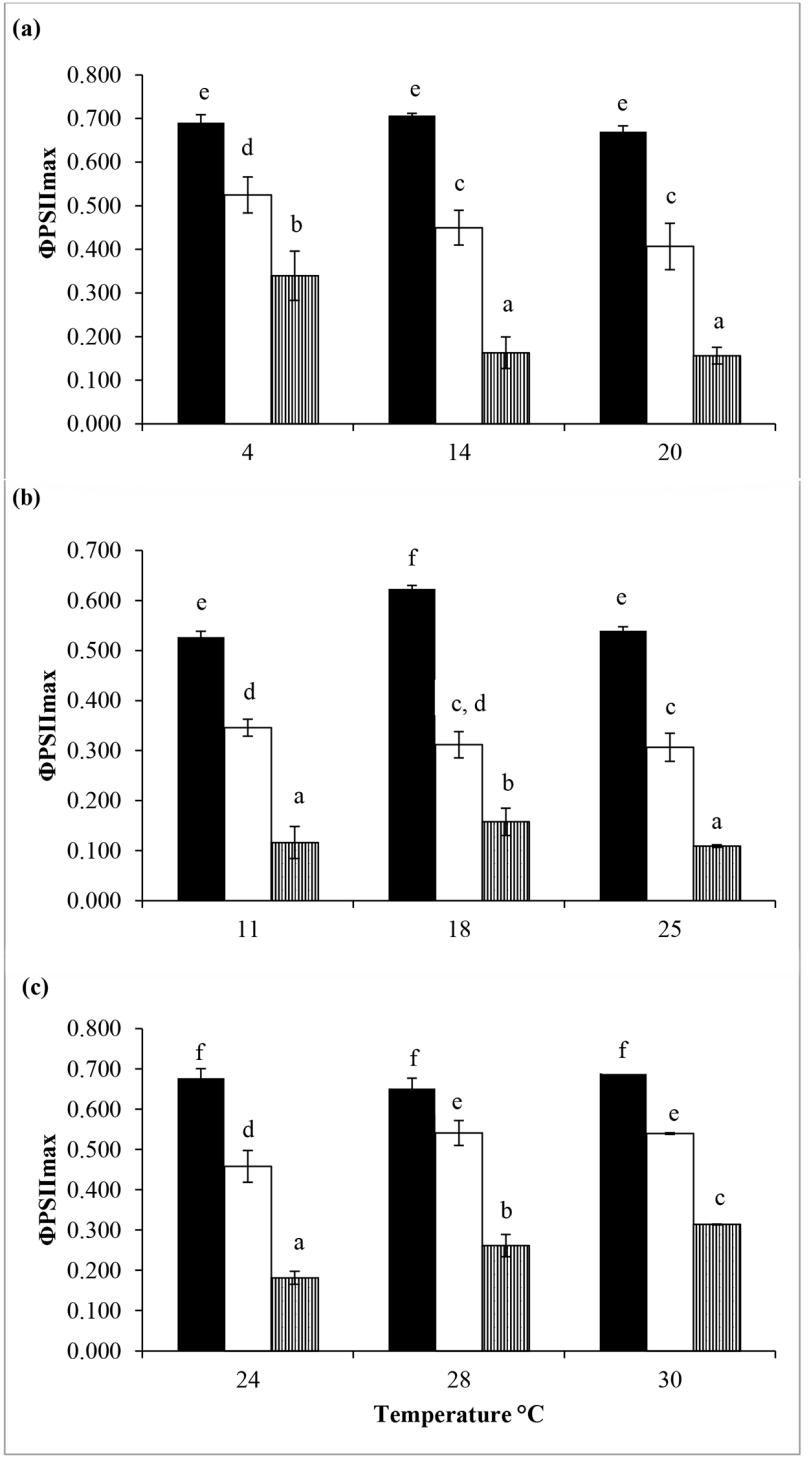
Effect of temperature and UVR on the maximum quantum yield of fluorescence (ΦPSII_max_) of (a) polar, (b) temperate and (c) tropical isolates of *Chlorella*. Vertical bars denote standard deviations from triplicate samples. Different letters indicate significant differences at p<0.05. PAR (filled), PAR + UV-A (open), PAR + UV-A + UV-B (hatched).

A general trend was observed in the effect of UVR on maximum quantum yield (ΦPSII_max_) of the three isolates of *Chlorella*. A significant reduction in ΦPSII_max_ was observed when the cultures were exposed to both PAR + UV-A and PAR + UV-A + UV-B compared to PAR alone (P<0.05) (Fig 1), with exposure to PAR+UVA+UVB causing maximum inhibition. However, isolates showed different trends in ΦPSII_max_ in their responses to increasing temperature. The effect of UVR on ΦPSII_max_ of both Antarctic and tropical *Chlorella* was temperature-dependent. The Antarctic *Chlorella* showed decreasing ΦPSII_max_ with increasing temperature under UVR exposure while the reverse trend was observed in the tropical *Chlorella* (Fig 1a). The ΦPSII_max_ values of the Antarctic *Chlorella* were 0.339, 0.163 and 0.156 when exposed to PAR + UV-A + UV-B at 4, 14 and 20°C, respectively. In contrast, the corresponding values of the tropical *Chlorella* were 0.181, 0.262, 0.314 when exposed PAR + UV-A + UV-B at 24, 28 and 30°C, respectively (Fig 1c). However, the reduction of ΦPSII_max_ in the temperate *Chlorella* under exposure to UVR was temperature-independent, whereby ΦPSII_max_ values at 11 and 25°C were significantly lower than that at 18°C (P<0.05) (ΦPSII_max_ = 0.116, 0.158 and 0.109 for 11, 18 and 25°C, respectively, under PAR + UV-A + UV-B) (Fig 1b).

**Fig 2:**
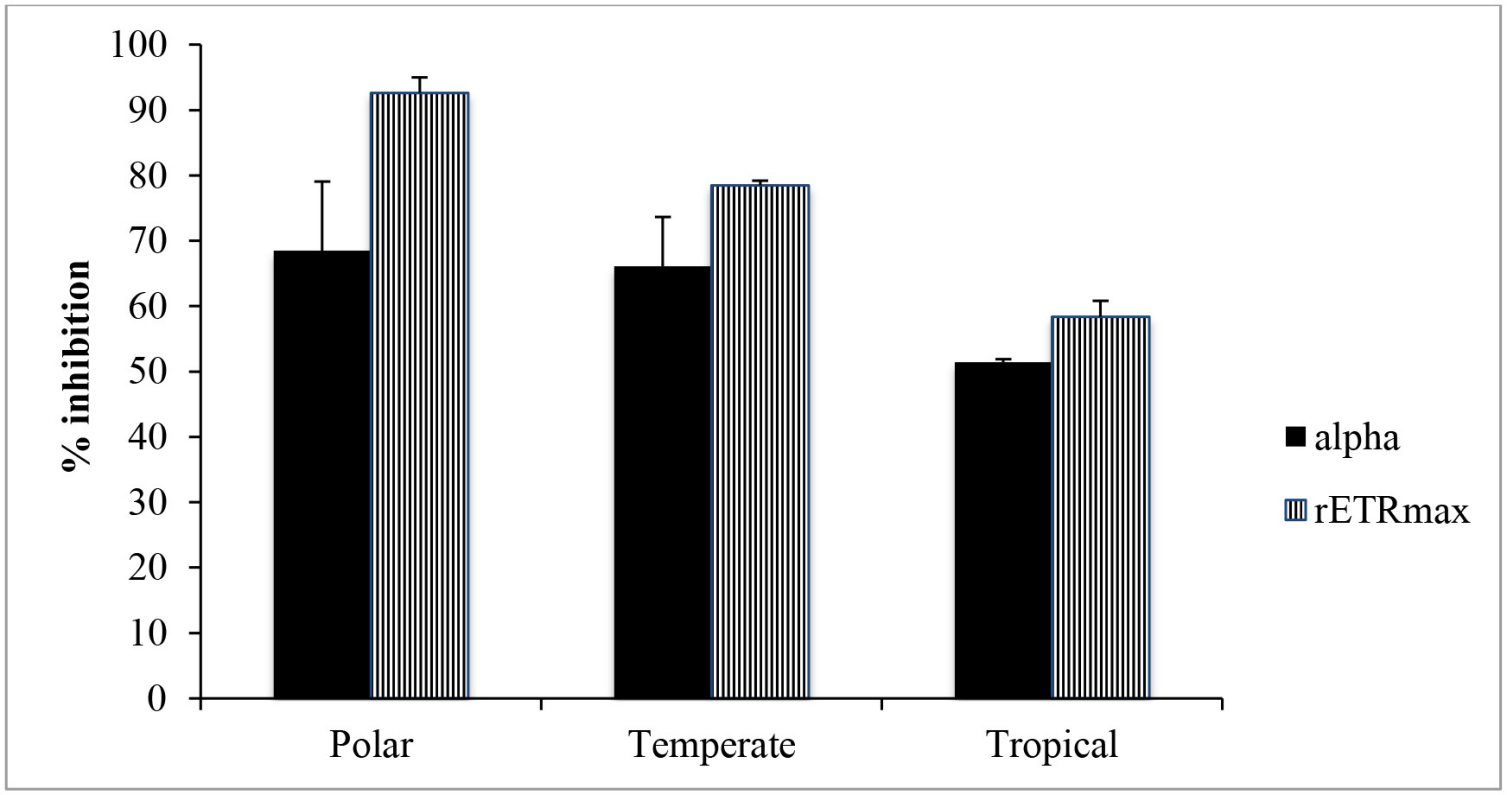
Inhibition of light harvesting efficiency (alpha) and maximal electron transport rate (rETR_max_) of polar, temperate and tropical *Chlorella* grown at their optimum temperatures (18, 20 and 30°C, respectively) then exposed to PAR + UV-A + UV-B for 60 min. Vertical bars denote standard deviations from triplicate samples.

Growth rates of Antarctic and temperate *Chlorella* have been shown to be optimal between 18 and 20°C, whereas the tropical isolate showed fastest growth at the maximum temperature (30°C) used in this study [35]. At these optimum temperatures, the photosynthetic characteristics, as represented by photosynthetic efficiency (alpha) and maximal electron transport rate (rETR_max_), of the tropical strain were less affected by UVR compared to the temperate and Antarctic strains (Fig 2).

**Fig 3:**
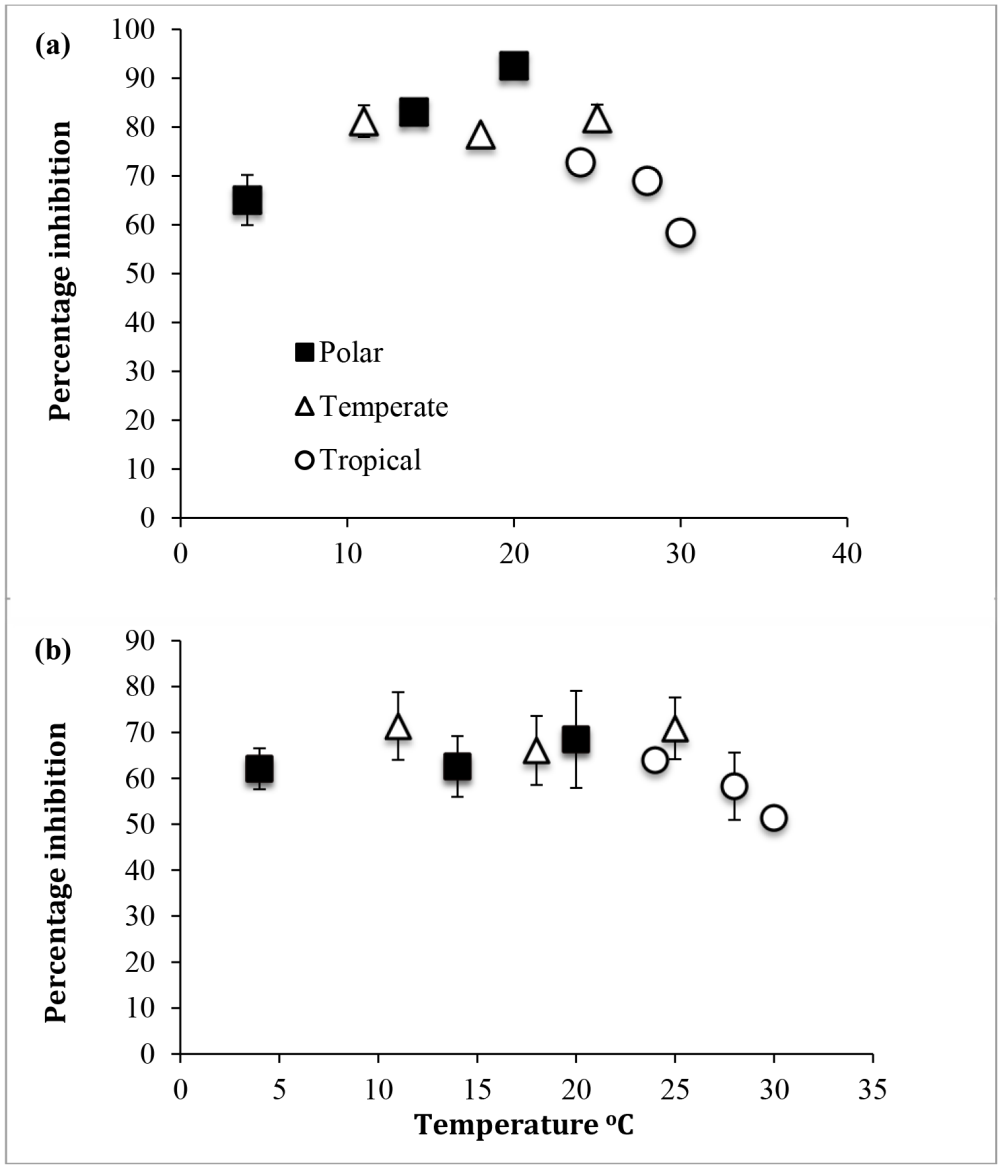
Percentage inhibition, by PAR + UV-A + UV-B, of (a) rETR_max_ and (b) alpha for all three isolates of *Chlorella*. Vertical bars denote standard deviations from triplicate samples.

The three isolates of *Chlorella* grown at different temperatures showed different trends in the inhibition of rETR_max_ and alpha by UVR (PAR + UV-A + UV-B treatment) (Fig 3). Inhibition by UVR of rETR_max_ for the Antarctic *Chlorella* showed a significant increase with increasing temperatures (P<0.05). The reverse trend was found in tropical *Chlorella*, and the temperate *Chlorella* showed no effect of temperature on UV-B inhibition of rETR_max_ (P>0.05). Inhibition of light harvesting efficiency (α) was essentially temperature independent except for the tropical strain at the higher temperatures used, which showed lower inhibition values (P<0.05).

**Fig 4:**
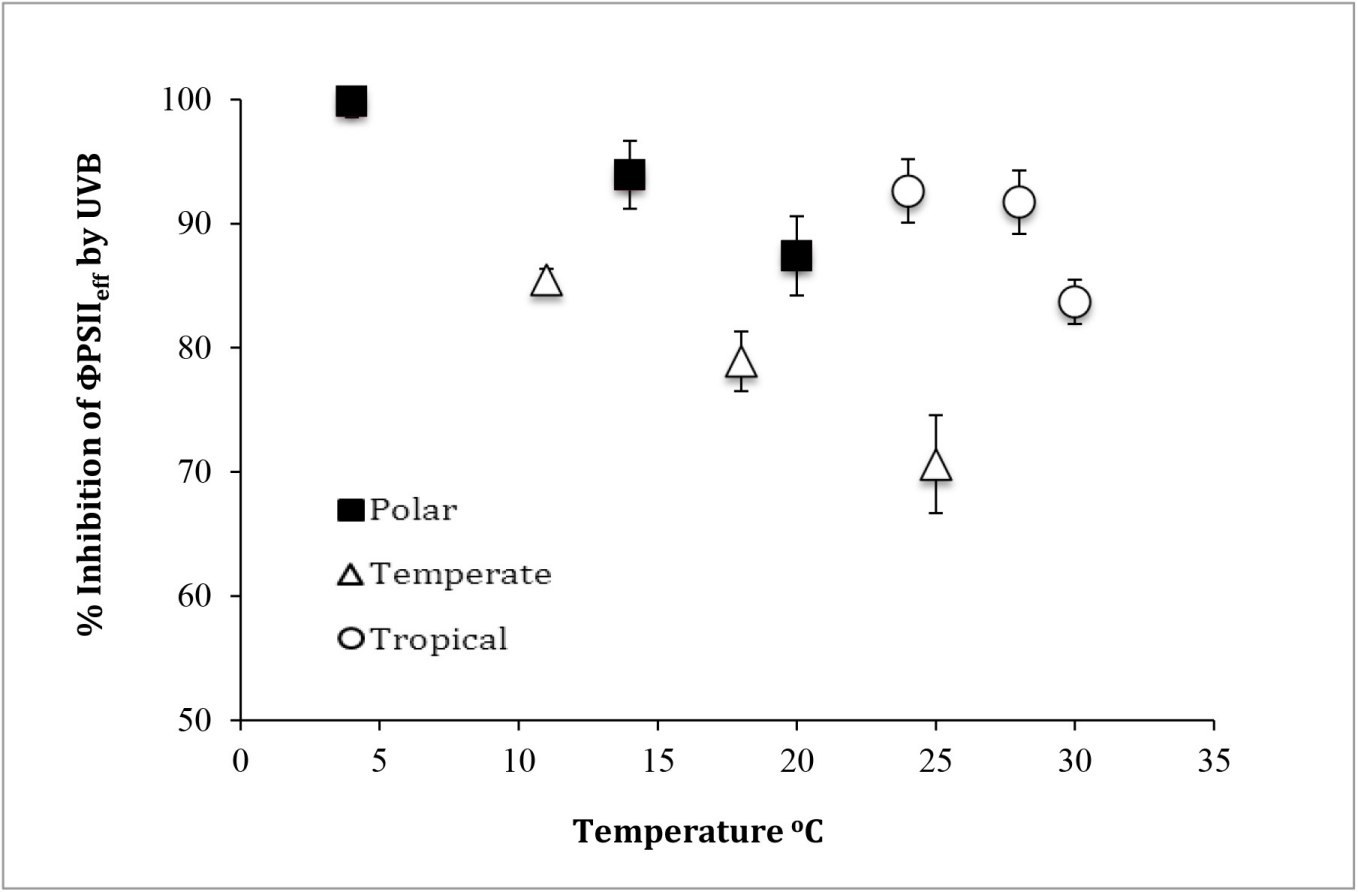
Inhibition of ΦPSII_eff_ of polar, temperate and tropical *Chlorella* after 60 min exposure to PAR + UV-A + UV-B. Vertical bars denote standard deviations from triplicate samples.

Cultures exposed to PAR + UV-A + UV-B showed a decline in ΦPSII_eff_, which fitted the Kok equation (Eq 1) well (data not shown). This allowed calculation of rate constants for damage (*k*) and repair (*r*) as well as the overall inhibition of ΦPSII_eff_ following 60 minutes of exposure to UVB, at which point all cultures had reached an asymptote and equilibrium levels of inhibition. Values for UVR inhibition of ΦPSII_eff_ after 60 min exposure were temperature dependent (Fig 4) with higher temperatures showing the least inhibition. This effect was most marked in the temperate strain and least marked in the tropical strain of *Chlorella*.

**Fig 5:**
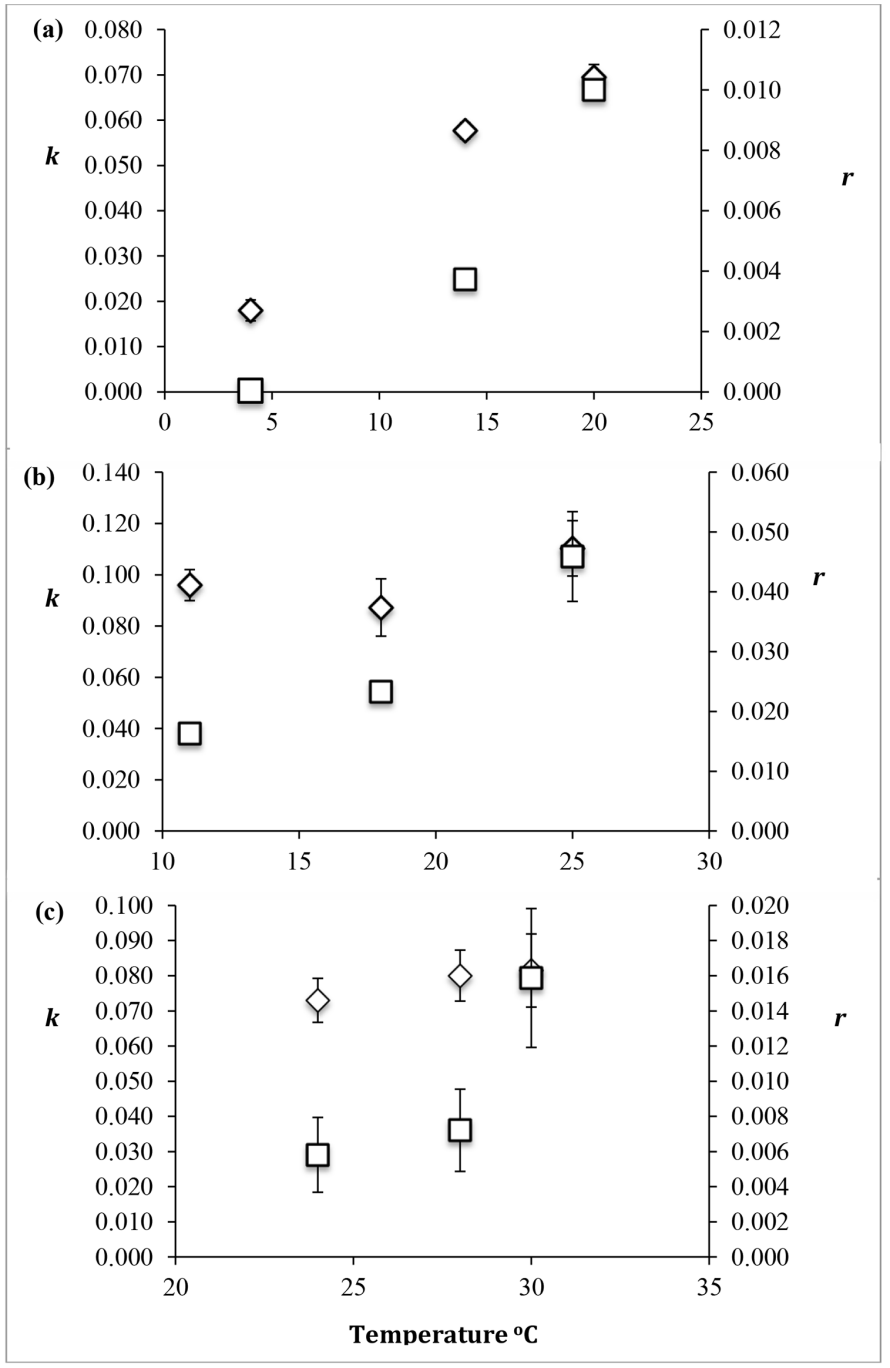
Damage (*k*, diamonds) and repair (*r*, squares) of (a) polar, (b) temperate and (c) tropical *Chlorella* strains as a function of temperature. Units for *k* and *r* are min^-1^. Vertical bars denote standard deviations from triplicate samples.

Although the rate constant for damage by UVR (*k*) was observed to increase with temperature in the Antarctic *Chlorella*, the repair constant (*r*) also showed an increase with temperature (Fig 5a). This indicated that the damage caused by UVR was being repaired faster at higher temperatures. For temperate *Chlorella*, damage from UVR was independent of temperature but the repair constant increased with increasing temperature (Fig 5b). In the tropical *Chlorella*, damage from UVR was unaffected by increasing temperature but repair strongly increased under higher temperatures (Fig 5c).

## Discussion

This study demonstrated the possibility of important interactions, between increased temperature associated with global change and the sensitivity of algae to UVR exposure, on the photosynthetic response of microalgae from different geographical regions. In general, there have been limited studies on the interactive effects of temperature and UVR on algae [38, 56, 57]. The interaction between these two factors can lead to variable responses. We hypothesized that UVR repair mechanisms are temperature dependent, while UVR-induced damage is less affected. Thus increasing global temperature might offset the negative effects of UVR due to increased repair or photoprotection at higher temperature.

In the present study, UV-A caused much lower inhibition than UV-B in photosynthesis in all *Chlorella* isolates, as shown by the value of maximum quantum yield, ΦPSII_max_. This is consistent with previous work that reported UV-A does not cause any adverse effect on the growth of Antarctic, tropical and temperate microalgae [31–33]. The low sensitivity of *Chlorella* isolates to UV-A could be due to the low UV-A radiation (and high UV-B:UV-A ratio) applied in the study. Nevertheless, UV-A has been shown to benefit photosynthesis of *Gracilaria lemaneiformis* [58] as well as that of phytoplankton species [59].

It is well known that exposure to high UV-B can cause detrimental effects on photosynthesis of algae [10, 11]. However, the three isolates of *Chlorella* examined here showed different trends in their photosynthesis responses under the combined effects of UVR (PAR + UV-A + UV-B treatment) and temperature. Of the three *Chlorella* isolates, the Antarctic strain showed a distinct decrease in ΦPSII_max_ when the culture was exposed to temperatures of 14°C and 20°C compared to 4°C. In addition, the percentage inhibition of rETR_max_ was highest for the cultures exposed to higher temperatures. This indicated that the UVR exposure resulted in photosynthesis stress, and that this stress increased with increasing temperatures, counter to our initial hypothesis that higher temperature would decrease UVR-sensitivity. This observation is, though, in accordance with the data on two sub-Antarctic brown algae, whereby elevated temperatures from 15 to 20°C exacerbated the detrimental effects of UVR on photochemical parameters such as ΦPSII_max_ and ETR [41]. However, the findings from this experiment are in contrast to those of van de Poll et al. [57] and Rautenberger & Bischof [38]. Both these studies reported that the photosynthetic efficiency of the Antarctic green algae *Ulva clathrata* and *U*. *bulbosa*, as well as the Arctic cold-temperate red algae *Palmaria palmata*, *Coccotylus truncatus* and *Phycodrys rubens*, was less affected by UVR at warmer temperatures. The increasing value of the damage constant (*k*) at higher temperature observed in the current study indicates that photosynthesis was badly affected under the combination of UVR exposure and increased temperature. This may be due to chronic inhibition of the photosynthetic apparatus or damage to key enzymes involved in photosynthetic production [60, 61]. The repair constant (*r*) increased with increasing temperature under UVR exposure and it may be postulated that the repair mechanism was temperature dependent. However, our results showed that the ΦPSII_max_ and rETR_max_ were negatively affected (more inhibited) by increasing temperature under UVR exposure. Therefore, it may be concluded that the repair (*r*) mechanisms at higher temperature are not sufficient to repair the damage caused by the UVR in the Antarctic *Chlorella* strain examined.

The photosynthesis response of the tropical *Chlorella* strain showed the reverse trend compared to the Antarctic strain. There was a negative effect of UVR on ΦPSII_max_ (decreased ΦPSII_max_ under UVR exposure compared to under PAR alone), however, the ΦPSII_max_ value increased significantly with increasing temperature. This was again supported by the observation that the lowest percent inhibition of rETR_max_ and alpha occurred when the temperature was increased to 30°C. Moreover, the repair constant (*r*) increased with the increasing temperature while the damage (*k*) was not affected by temperature. This is consistent with the concept that elevated temperatures in tropical environments are conducive to high rates of UV-B tolerance and is in agreement with the finding of Gao et al. [39] who reported that the inhibition of photosynthesis (decrease in effective quantum yield) due to both PAR and UVR decreased in *Arthrospira platensis* when the temperature was increased from 15 to 30°C. The increasing value of the repair constant (*r*) at higher temperature is consistent with the faster turnover of the D1 protein that led to recovery, after UVR treatment, of desmid strains from different geographical regions as demonstrated by Stamenković & Hanelt [52]. These authors speculated that the tropical species *Cosmarium beatum* displayed exceedingly high rates of *de novo* protein synthesis after UVR treatment under a higher temperature, which was in accordance with the fact that plant and algal species adapted to high-light intensities possess exceedingly high rate of D1 turnover and *de novo* protein synthesis [52, 62, 63]. In addition, UVR-induced photoinhibition of Photosystem II was less pronounced in sporophytes of the red alga *Gelidium pulchellum* at 20°C compared to 15°C [56]. These authors speculated that repair and photoprotective mechanisms under UVR were equally stimulated by increasing temperature, results supported in part by direct measurements of *r* and *k* in the present study. Hoffman et al. [36] reported that the inhibitory effect of UVR on spores and gametophytes of *Alaria marginata* as well on zygotes and germlings of *Fucus gardneri* was less strong at higher temperatures.

The optimum growth temperature for the temperate *Chlorella* strain is 18°C [35]. Increasing or decreasing the temperature, in combination with UVR exposure, relative to this optimum caused a slight decrease in ΦPSII_max_, while the percent inhibition of rETR_max_ and alpha by UV-B was not significantly different to those at its optimum temperature. As for the other strains, damage was unaffected by temperature but repair was stimulated.

Although the photosynthetic apparatus of the *Chlorella* strains examined here was severely inhibited by the acute UVR dose applied in this study, the damage constant was less affected by temperature than repair implying that algae would be advantaged, in terms of UV-B sensitivity, by elevated temperatures resulting from global change. This is consistent with observations that higher temperatures decrease sensitivity of some algae to UVR ([64] and references therein)

Tolerance to UVR can be due to various mechanisms in addition to repair processes; these include ROS quenching, increase in antioxidative stress compounds as well as production of photoprotective compounds such as mycosporine amino acids (MAAs) and carotenoids. Although our data suggest that phototrophic organisms living in cold environments may be especially prone to the damaging effects of UVR because of the limited repair capabilities at low temperatures [65] and that higher temperatures may ameliorate the damaging impact of UV-B, effects of temperature on these other processes need to be taken into account. Furthermore, increased surface temperatures in water bodies (oceans, lakes) will, as a consequence of enhanced stratification lead to decreased nutrient availability and higher PAR as well as UV-B fluxes, which also impact on the sensitivity of algae to UV-B [25]. The interplay of these factors is likely to be complex, so a clear picture of how algae will respond will require considerable future study of these interactions.
